# Microbial Polymers as Sustainable Agents for Mitigating Health Risks of Plant-Based Endocrine Disruptors in Surface Water

**DOI:** 10.3390/ijerph181910040

**Published:** 2021-09-24

**Authors:** Moushumi Ghosh, Krishnaswamy Balamurugan, Vivek Sharma

**Affiliations:** 1 Department of Biotechnology, Thapar Institute of Engineering and Technology, Patiala 147004, Punjab, India; vivek.microbio@gmail.com; 2 Department of Biotechnology, Alagappa University, Karaikudi 630003, Tamil Nadu, India; bsuryar@yahoo.com

**Keywords:** phytoestrogens (PE), biopolymers, *Caenorhabditis elegans*, adsorption, toxicity, surface water

## Abstract

This study investigated the binding abilities of extracellular polymers produced by an environmentally isolated strain of *Enterococcus hirae* towards phytoestrogen endocrine disruptors—biochanin A, formonetin, genistein and daidzein. The extracellular biopolymer exhibited notable binding and removal for all four phytoestrogens, with a maximum removal of daidzein (87%) followed by genistein (72%) at a 1–1.5 mg/mL concentration. Adsorption proceeded rapidly at ambient temperature. The adsorption data fitted well with the Langmuir isotherm. Based on the adsorption energy, the biopolymer binding of phytoestrogens was inferred as daidzein > genistein > biochanin A > formononetin. Toxicity of the biopolymer (5–250 µg/mL) evaluated using RAW 264.7 cell lines indicated no significant (*p* < 0.05) changes in viability. In biopolymer-challenged *Caenorhabditis elegans* previously exposed to daidzein, complete protection to developmental toxicity, such as reduced egg-laying capacity, egg viability and progeny counts of the worm, was observed. The results of this study offer valuable insights into understanding the potential role of microbial extracellular biopolymers in binding and removal of phytoestrogens with sustainable technological implications in modulating the toxic effect of high levels of endocrine disruptors in the environment.

## 1. Introduction

The presence of man-made and naturally occurring contaminants in aquatic ecosystems has received increased attention as human activities continue to introduce complex mixtures of contaminants into surface waters. Both agricultural practices and industrial discharges are key contributors of endocrine-disrupting materials. In areas where agricultural practices are intense, endocrine disruptors from plant sources or phytoestrogens are prevalent in surface water. For instance, crop residues and wastes after harvest remain in fields for sufficiently long periods of time and subsequently start rotting after rainfall, and then the leachate directly runs off into water bodies.

Frequently, crop residues are directly discarded in or near water bodies, which end up in water. These two practices are widely followed and little or no investigations of the surface water or sludge have been carried out, assuming agro practices do not generate hazardous wastes. In a previous study, we sampled 145 such areas, indicating an abundance of phytoestrogens in these areas. These areas are possible hotspots. The leachate water drains into rivers, streams and canals; the latter serving as drinking water to animals and humans in adjoining villages. Phytoestrogens were detected also in tube wells and wells in 17 locations of the villages surveyed. Furthermore, wood industries and agro processing industries are important contributors of phytoestrogens in surface water. Phytoestrogens are naturally occurring plant compounds that are structurally or functionally similar to mammalian oestrogens and their active metabolites. Among them, lignans, isoflavones, flavonoids and coumestans form the major classes [1]. Many isoflavones have the ability to inhibit cell proliferation and growth and interfere with the organizational role of oestrogens in developing brain and reproductive systems [2]. Manipulation of the oestrogen level during critical development phases, such as gestation and early infancy, leads to adverse health outcomes, including malformations in the ovary, uterus, mammary gland and prostate, early puberty, reduced fertility, disrupted brain organization and reproductive tract cancers [2]. Presence of phytoestrogens in lakes and other water bodies have a significant effect of inducing a skewed sex ratio in fishes, resulting in a gross decline in productivity. To date, several reports have clearly indicated the potential loss of fish productivity upon exposure to phytoestrogens and synthetic endocrine disruptors can have potential sublethal effects on aquatic life, especially during early life stages. The continuous exposure is pertinent when sexual development is most sensitive [3]. Thus far, phytoestrogen removal from water has resorted to ultrafiltration, reverse osmosis photodegradation, membrane retention and adsorption. However, competing substances in water streams, such as natural organic matters (NOMs), influence the adsorption of these compounds and, as a result, the removal efficiency for the target compounds can be adversely influenced [4]. Biological treatment, which is the most important treatment process of wastewater, and has been studied extensively by various researchers, is ineffective in removing these compounds [5].

Recently, enzyme- and antibody-based approaches have been suggested, although effective the specificity, operational requirements and cost limitations have restricted their field application [6]. Therefore, there is a critical need for sustainable mitigation methods for the increasing levels of phytoestrogens in surface waters. Renewable, natural sources with effective phytoestrogen-binding ability can be appropriate agents for sustainable treatment options. Microbial extracellular polymeric materials are currently considered as green material with multiple and diverse functionalities. In the recent past, a significant volume of research has been dedicated in elucidating microbial extracellular polymers for environmental remediation. However, studies focusing on phytoestrogen binding and subsequent approaches for removal from water remain largely unexplored. In the present study, we evaluated the extracellular polymers elaborated by bacterial isolates from phytoestrogen-rich sites. Based on the phytoestrogen-binding efficacy, *E. hirae* was selected from amongst the isolates and investigated as a potential strain for biopolymer production and phytoestrogen removal studies in vitro. Alleviation of the phytoestrogen (PE) toxicity in the worm *Caenorhabditis elegans* was subsequently evaluated. We envisaged that insights from the study will be important for developing interventions to reduce waterborne phytoestrogen endocrine disruptors, especially in areas where intense agricultural operations and plant processing industries are abundant.

## 2. Materials and Methods

### 2.1. Microorganisms and Biopolymer Production

The bacterial isolates *Bacillus cereus*, *Citrobacter brakii* MG303, *Bacillus cytotoxicus* MG303, *Enterococcus vikkiensis* and *Enterococcus hirae* MG6, previously characterized from the vicinity of phytoestrogen-rich wastewater and soil sites, were used to obtain extracellular biopolymers with phytoestrogen-binding abilities. The phytoestrogens biochanin A (BCA), genistein (GE), formonetin (FM) and daidzein (DAZ) (Sigma, MO, USA) were used for the study.

All bacterial strains were grown overnight in FIB (Flocculant Induction Medium) at 30 °C with shaking; biopolymer production and purification was carried out as described by [7] (Khaira et al. (2014)).Initially, purified biopolymers from each strain were analysed with the four PEs. Since the extracellular biopolymer produced by *E. hirae* exhibited the highest phytoestrogen-binding capacity, this strain was finally selected for biopolymer production and further studies. The selected bacterial culture was grown overnight in FIB, the supernatant centrifuged and the biopolymer precipitated by the addition of two volumes of ethanol for precipitation at 4 °C for 24 h. The precipitated biopolymer was collected by filtration (Whatman GF Filter, Bedfordshire, UK), dissolved in deionized water and dialyzed extensively against deionized water. The biopolymer was further dissolved in deionized water and re-precipitated by adding a 10% solution of cetylpyridinium chloride (CPC). The precipitated complex was collected by centrifugation at 10,000× *g* for 20 min at 4 °C and redissolved in a 10% NaCl solution, and finally recovered by addition of three volumes of ethanol. The extracted biopolymer was then dissolved in deionized water, dialyzed twice against deionized water and lyophilized.

### 2.2. Biopolymer Characterization and Adsorption Studies

The *E. hirae* biopolymer was characterized chemically for its constituents and structural linkages by Fourier transformed infrared spectroscopy (FTIR). Its molecular mass was determined by gel permeation chromatography (GPC). Morphological characterization was carried out by scanning electron microscopy (SEM) (Sharma and Ghosh, 2021) [8].

The *E. hirae* biopolymer (1 mg/mL) and phytoestrogens (standard solution, i.e., 1 µg/mL) were mixed, vortexed for 1 min and then shaken manually for some time in 250 mL Erlenmeyer flasks. Solutions used were 50 mL, with the respective phytoestrogens separately, and agitated at a constant speed of 130 rpm at a temperature of 28 °C.

At the intervals of 15, 30, 45 and 60 min, 1 mL of these samples were withdrawn and spun down at 10,000 rpm for 1 min at room temperature. Absorbance of the supernatant was recorded at the maxima of the respective phytoestrogens [9]. Adsorption capacity was determined by Langmuir’s adsorption isotherm [10]. Biopolymer at equilibrium (Q_e_, mg/g) was calculated by using the following equation:

Q_e_ = (C_o_V_o_ − C_e_V_e_)/m

where C_o_ and C_e_ are the initial and final sample absorbance before and after treatment, respectively; V_o_ and V_e_ are the initial and final volume of the sample solution; and m is the mass of biopolymer added. The plot of C_e_/Q_e_ vs. C_e_ is a straight line with a slope of 1/Q_max_ and intercept of 1/Q_max_K_L_. The monolayer (maximum) adsorption capacities (Q_max_) obtained from the Langmuir plots give the idea of effectiveness of the adsorbents towards the adsorbate.

### 2.3. Biopolymer Toxicity

Biopolymer toxicity was evaluated in murine macrophage (RAW 264.7) cell lines procured from the National Centre for Cell Science (NCCS), Pune, India. RAW 264.7 were maintained in complete RPMI (Rosewell Park Memorial Institute Medium) in a humidified incubator with 5% CO_2_ at 37 °C. For assaying toxicity, cells were seeded in 96-well plates at a density of 1 × 10^4^ cells/well. The purified *E. hirae* biopolymer was added at the concentrations of 5, 10, 15, 20, 25, 50, 75, 100, 150, 200, and 250 µg/mL, respectively, in the culture medium for 24 or 48 h. The cell-surviving rate was quantitated by MTT assay by adding 20 µL of 5 mg/mL MTT to each well followed by incubation for 3 h. The supernatant was removed and 200 µL DMSO (dimethyl sulfoxide) was added to each well. The absorbance of each well was measured (570 nm) using a microplate reader.

### 2.4. C. elegans Culture 

*Caenorhabditis elegans* (*C. elegans*) N2 WT strain were obtained from CGC (Caenorhabdtis Genetics Center) funded by the National Institutes of Health, National Center for Research Resources, and maintained in Nematode Growth Media (NGM) seeded with *E*. *coli* OP50 as the nutrient source and maintained at 20 °C [11]. Age-synchronized young adult were used for the study. The synchronization of worms was performed through bleaching the group of gravid hermaphrodites using a 5M alkaline hypochlorite solution and commercial bleach in the ratio of 1:111. The experiments were performed in triplicates. 

#### 2.4.1. Dosage Fixation and Survival Assay

Batches of 10 age-synchronized *C. elegans* at young adult stage were introduced into a 24-well plate containing M9 buffer along with *E. coli* OP50 as a food source. The phytoestrogens dissolved in methanol were administered at various concentrations (50–250 µg/mL phytoestrogens). As a control, *C. elegans* was administered with either methanol alone or M9 buffer. Readings were recorded for 70 h at 20 °C at an interval of 2 h; worms were considered to be dead if they did not respond to a gentle touch by using a platinum worm picker.

In vivo assessment and microscopic analysis of *C. elegans* treated with phytoestrogens (PE): Synchronized young adult worms were exposed with phytoestrogen (PE concentration—50 µg/mL dissolved in water and DMSO in case of BCA) and incubated at 20 °C in a 24-well plate with *E.coli* OP50 as diet. DMSO was used as the vehicle control for BCA. After every 8 h of incubation, the worms were monitored for cytotoxic effects. To score for worm survival, the plates were shaken by hand and the worms were considered dead if they did not move or exhibit pharyngeal movement. Further confirmations of the dead worms were made if they did not respond to touching with a platinum wire pick and did not show any pharyngeal movement for several hours. After 24 and 48 h of incubation, the worms were microscopically monitored for spatiotemporal effect using a Nikon Eclipse camera (Japan) attached to a bright-field microscope (80i) (Zeiss, Oberkochen, Germany).

#### 2.4.2. In Vivo Assessment and Microscopic Analysis of *C. elegans* Treated with Biopolymer

Synchronized young adult worms were infected with purified *E. hirae* biopolymer (concentration used was 50 µg/mL) and incubated at 20 °C in 24-well plates with *E. coli* OP50 as diet. After 24 and 48 h of incubation, the worms were microscopically monitored for spatiotemporal effect using a Nikon Eclipse camera attached to a bright-field microscope (80i).

#### 2.4.3. In Vivo and Microscopic Analysis of Combinatorial Effects on *C. elegans* Treated with Phytoestrogen Followed by Biopolymer & Vice Versa

Synchronized young adult worms were pre-treated with PE and after 24 h with biopolymers (Vice versa) incubated at 20 °C in 24-well plates with *E. coli* OP50 as diet. After every 8 h of incubation, the worms were monitored for cytotoxic effect. To score for worm survival, the plates were shaken by hand and the worms were considered dead if they did not move or exhibit pharyngeal movement. Further confirmations of the dead worms were made if they did not respond to touching with a platinum wire pick and did not show any pharyngeal movement for several hours. For microscopic analysis, synchronized young adult worms were pre-treated with PE and after 24 h with the biopolymer (Vice versa) incubated at 20 °C in 24-well plates with *E. coli* OP50 as diet. After 48 h of incubation, the worms were microscopically monitored for spatiotemporal effect using a Nikon Eclipse camera attached to a bright-field microscope (80i). An egg count assay was carried out by the method described by Harlow et al. (2016) [12].

### 2.5. Statistical Analysis

All experiments were performed in triplicates and data were represented in the form of the mean and standard deviation. Fisher’s exact test and one-way ANOVA were used for assessing survival assay, egg-laying capacity, egg count and progeny count between the vehicle control and indigenous samples. PEs was considered to be toxic if the mean number of eggs and progeny in response to the given dose was significantly different than the control vehicle or the number of unhatched eggs was significantly higher (*p* < 0.001).

## 3. Results

Prior to this study, the extracellular biopolymers from *Enterococcus hirae* MG6, *Bacillus cereus*, *Citrobacter brakii* MG303, *Bacillus cytotoxicus* MG303 and *Enterococcus vikkiensis* were evaluated for their binding ability to PEs (phytoestrogens: daidzein, genistein, biochanin A and formononetin) (Table 1).

**Figure 1:**
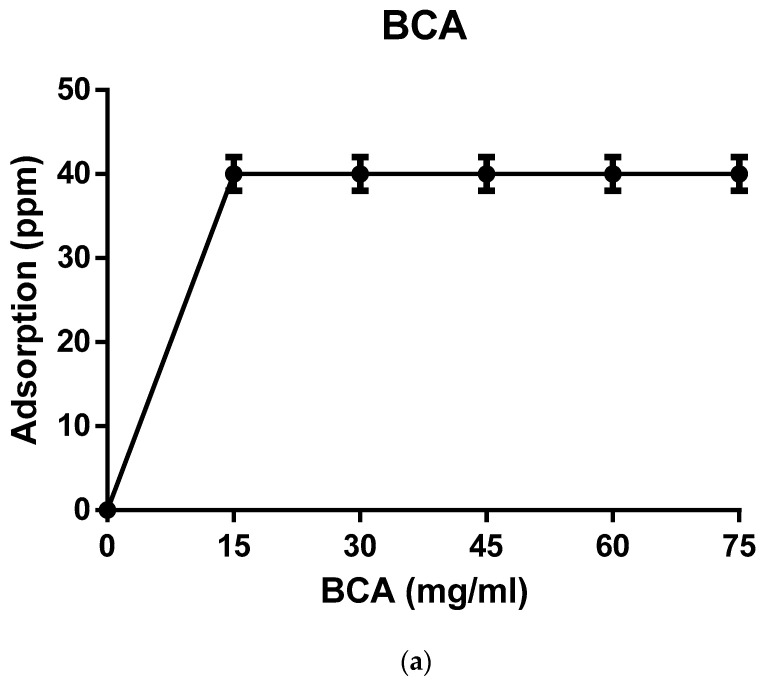

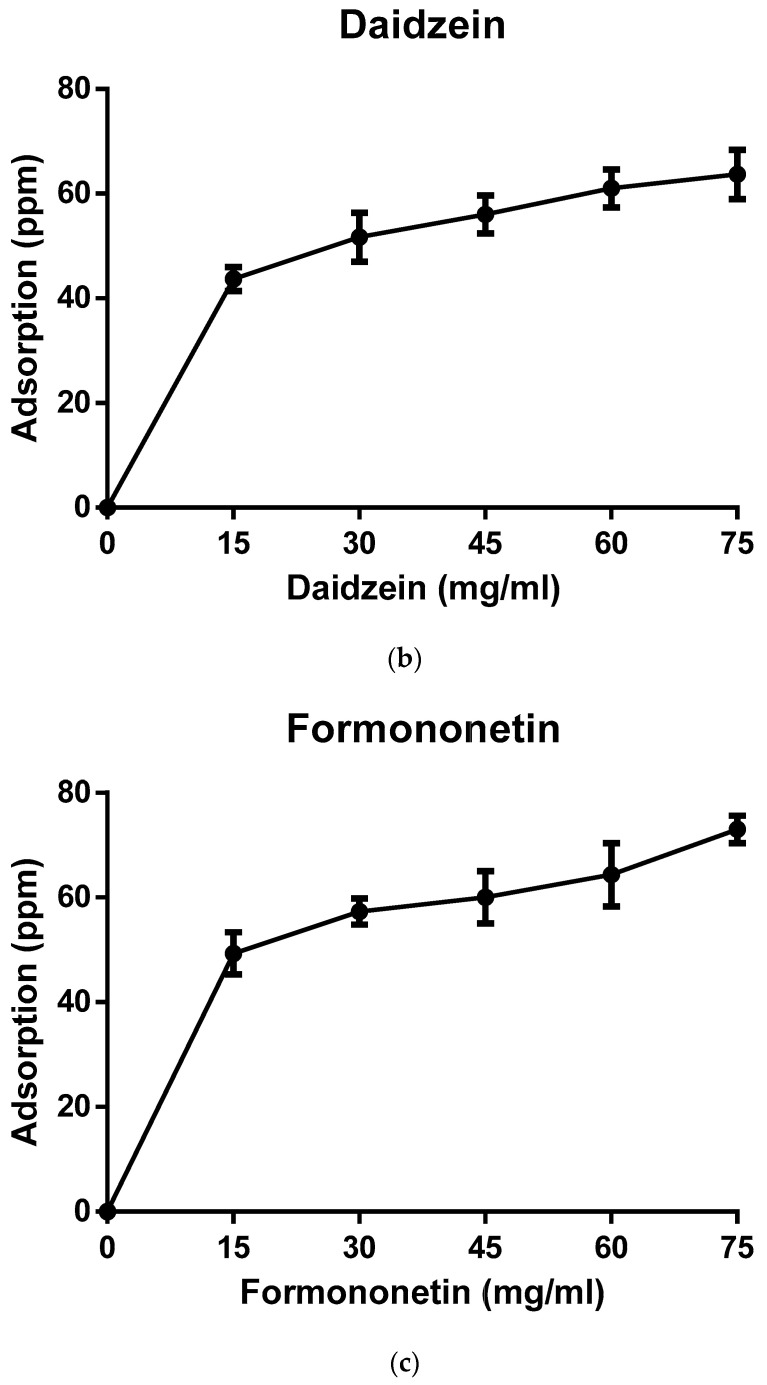

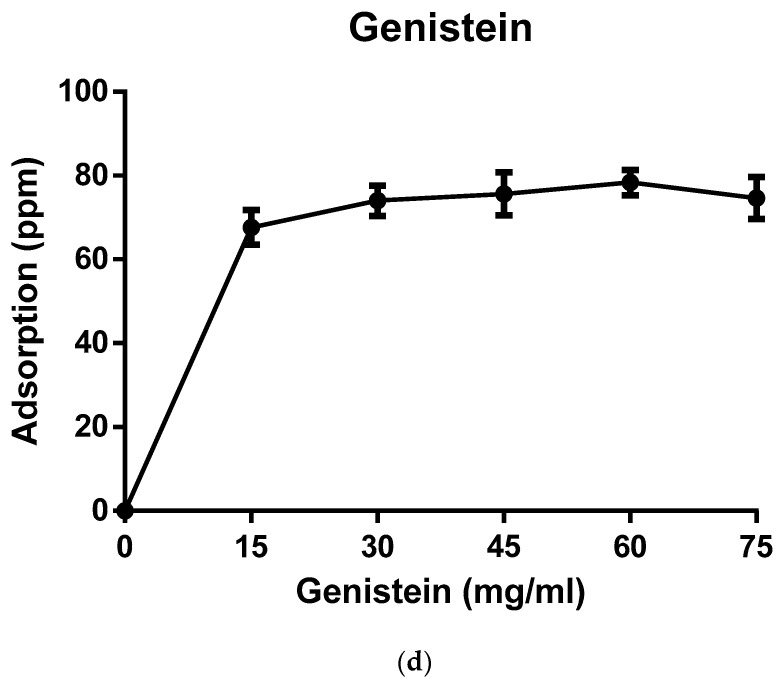
Effect of biopolymer contact time on the adsorption of phytoestrogens: (**a**) formononetin, (**b**) genistein, (**c**) biochanin A, and (**d**) daidzein. The error bars represent the ±SD of three replicates.

Based on its high phytoestrogen-binding ability, the *E. hirae* biopolymer was selected for this study. Binding of phytoestrogens remained unchanged after 30 min at ambient temperature. The reduction percentage of daidzein was the maximum (87%) while the percentage reduction of genistein was 72% (Figure 1). The molecular mass of the *E. hirae* biopolymer was found to be 2 × 10^5^ kDa. Chemical analysis indicated carbohydrates (38%) to be the principal component followed by hexosamines (26%) pyruvic acid uronic acids. Lipids were not detected while the protein concentration was 18.6% (Supplementary Table S1). FTIR signatures revealed prominence of aliphatic, aromatic and amino groups. Scanning electron micrographs indicated porosity and a compact structure (results not shown).

**Figure 2:**
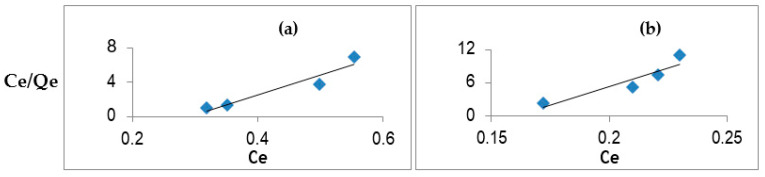
Langmuir adsorption isotherm for (**a**) daidzein and (**b**) genistein. Mean values obtained from three replicates are shown. Among the isoflavones, i.e., D, F, GE and BCA, and the Q_max_ value of the biopolymer, the saturation concentration, i.e., 1 mg/mL and 1.5 mg/mL, was found to be in the following order: daidzein > genistein > biochanin A > formononetin, respectively. As K_L_ is related to the energy of the adsorption process, a trend was generated based on the adsorption energy of the phytoestrogens and can be inferred as daidzein > genistein > biochanin A > formononetin.

The Langmuir plot of C_e_/Q_e_ against C_e_ exhibited good linearity (R^2^ = 0.95 to 0.99), indicating that adsorption of the phytoestrogens obeys the Langmuir adsorption isotherm (Figure 2). The values of K_L_ and Q_max_ of the *E. hirae* biopolymer are depicted in Table 2. It was found that the biopolymer that has greater adsorbent capacity Q_max_ has a higher value of K_L_, and vice versa. Higher values of K_L_ represent an effected adsorption of phytoestrogens [13]. The correlation coefficient values (R^2^) showed good linearity to the biopolymer and phytoestrogens. Thus, the Langmuir adsorption isotherm fits well to these adsorption studies.

Exposure of the biopolymer to RAW264.7 cell lines, at different doses (Table 3), indicated no significant (*p* > 0.05) decline in cell viability over 24 and 48 h exposure for the range of biopolymer doses (5–250 µg/mL) tested, suggesting the safety of the biopolymer.

The worms exposed to phytoestrogens in the survival assay, indicated as 80%, 80%, 60% and 40% mortality at 70 h, respectively, in comparison to the vehicle control, suggest the toxicity of biochanin A, formononetin, genistein and daidzein to the worms during prolonged exposure. The difference in the progeny count between the exposed groups and the vehicle control on Days 2 and 4 was significantly (*p* < 0.05) low. The lethal concentration of *C. elegans* to PEs after 24 h is shown in Table 3b.

**Figure 3:**
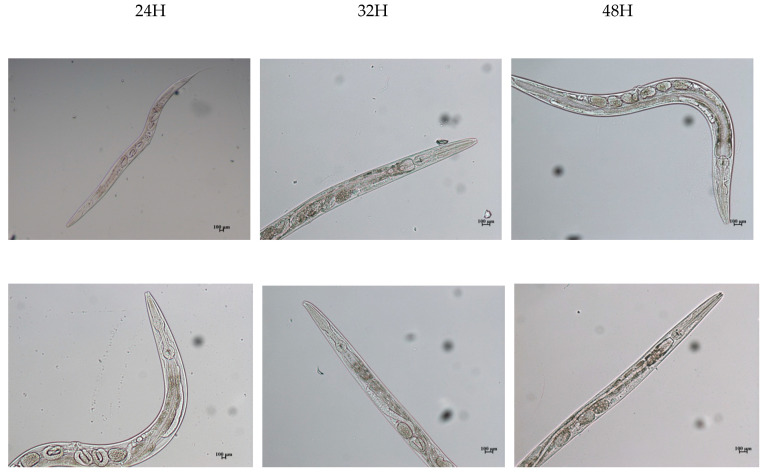
Status of model animal, *C. elegans*, during exposure studies. Young adult *C. elegans* were exposed to PEs and the impact on reproduction/development was compared with the controls. Representative images showing the spatiotemporal (20×) effect of biochanin A (**bottom panel**) on the model animal, *C. elegans*, when compared to the control (**top panel**) OP50 (food source).

Based on these observations, a sub lethal concentration of 50 µg of phytoestrogens was used throughout. Microscopic analysis for spatiotemporal effects of PE-challenged *C. elegans* revealed potential toxic effects. The egg-laying capacity of biochanin A-treated *C. elegans* was similar either in the presence or absence of the biopolymer treatment. Hence, the biopolymer treatment was ineffective for *C. elegans* treated with biochanin A (BCA) (Figure 3). In formonetin (FM)-treated *C. elegans*, eggs were not produced. The biopolymer from the *E. hirae* strain MG6 was partially effective in restoring egg production, as observed by the distorted eggs (results not shown).

**Figure 4:**
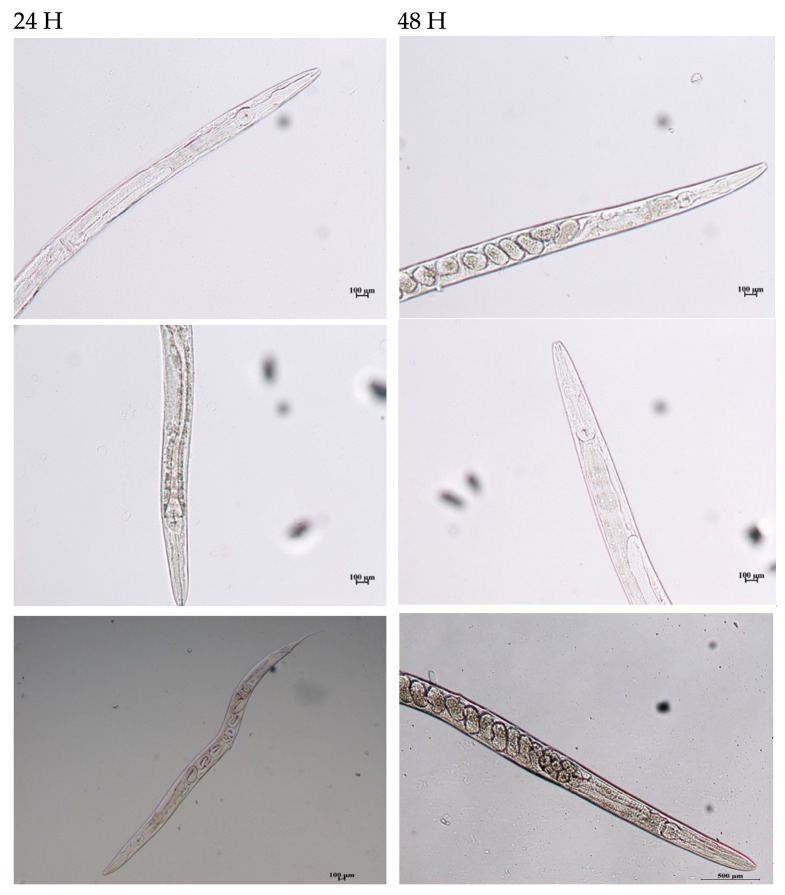
Status of model animal, *C. elegans*, during biopolymer treatment. Young adult *C. elegans* exposed to biopolymers and the impact on reproduction/development was compared with the controls. Representative images showing the spatiotemporal (20×) effect of the *E. hirae* biopolymer on the model animal, *C. elegans*, when compared to the control OP50 (food source, bottom Left (24 H) and Right (48H).In the biopolymer-treated animals exposed to daidzein, delayed production of eggs was initially observed but subsequently normalcy was restored.

When *C. elegans* was treated with daidzein, premature eggs were produced; addition of biopolymer (50 µg) restored the normal life cycle of the worm (Figure 4). It is possible that higher concentrations of the biopolymers can exert the desired removal and reversal of toxicity of the other phytoestrogens. The relatively smaller molecular size of daidzein could be a possible reason for this observation where a complete binding and alleviation of toxicity occurred at a 50 µg biopolymer concentration. Though the biopolymer was tested for toxicity, for all the other phytoestrogens there was no alleviation of toxicity, indicated by an abnormal life cycle and egg-laying capacity. Nevertheless, the results reflect the potential applicability of the *E. hirae* biopolymer in removal of waterborne phytoestrogen endocrine disruptors, which is important for reducing the toxicity.

## 4. Discussion

Surface water is an important dumping ground for both synthetic and natural endocrine-disrupting molecules. For instance, in an earlier study we reported high quantities of bisphenol A leaching from plastic wastes within city premises [13]. In several earlier studies, high levels of phytoestrogens (biochanin A, formonetin and daidzein) were detected near a paper industry, agro industries and farms (145 µg/mL). Nanozeolites and activated carbon-impregnated biopolymers were found effective for removing the endocrine disruptors. Recently, a magnetic multiwall carbon nanotube modified with chitosan was successfully prepared and applied for adsorption of bisphenol A (BPA) from aqueous solutions [14]. Oestrogens and β blockers from water were effectively removed using biomass from activated sludge by Gabet-Giraud et al. (2010) [15]. However, few works have exploited the structural diversity of microbial polymers. Therefore, a systematic screening was initially carried out for understanding the binding efficacy of phytoestrogens to the biopolymers, elaborated by selected bacterial isolates. These isolates were screened from sites with a history of phytoestrogens. In the present study, nearly 10 to 50% of the total phytoestrogen reduction appeared to have been adsorbed by the purified *E. hirae* biopolymer in the first 30 min, and at equilibrium it reached up to 75 to 80%, depending upon the adsorption ability of the biopolymer. It has been suggested that the rapid adsorption at the initial stage of adsorption was because of a higher number of active sites on the surface of the adsorbent. A strong binding is imperative for preventing the toxic effects of phytoestrogens in *C. elegans*, which can be easily interpreted by the developmental assay. The latter model was chosen because unlike in vitro testing, *C. elegans* toxicity assays provide data from an animal with intact and metabolically active reproductive, digestive, sensory, endocrine and neuromuscular systems. Features such as a small size, short life cycle, ~80% homology to human genes, ease of obtaining synchronized isogenic populations and simplicity of maintenance and culturing make it a powerful and established model for high-throughput screening [16]. Convenient biomarkers, such as fecundity, can enable *C. elegans* as an effective model organism for studying the chemical actions that lead to the disruption of reproduction [17].

Mortality and other physiological parameters, such as development, locomotion and reproduction, provide a vivid picture on the impact of toxicity on the *C. elegans*. Disruption in events such as egg laying and progeny production affords an understanding of the extent of damage caused due to the toxic effects of the phytoestrogens.

All four phytoestrogens were potentially toxic to *C. elegans*, with adverse implications on its survival and reproductive behaviour in terms of egg-laying capacity and egg viability.

The physiological parameters selected for toxicity analysis of samples were egg-laying capacity and progeny count analysis (developmental toxicity), where a chemical adversely affects the biological processes of development from egg to adult [17]. These effects are demonstrable in assays with selected phytoestrogens but lacking a biopolymer. However, the strong affinity of the *E. hirae* biopolymer with phytoestrogens did not allow manifestation of toxicity, which was the case for daidzein. The mechanism of in vitro adsorption of the phytoestrogens with biopolymers was similar to those reported [18]. The surface properties of the biopolymer, concentration and nature of phytoestrogen may be attributed to the differential adsorption capacities. Simulated studies using surface water containing spiked phytoestrogens indicated a similar trend (unpublished observations). Specific interactive effects of other waterborne components on biopolymer adsorption performance, however, require detailed studies. Since surface water is home to phytoestrogens, occurring from sources such as agro-processing, paper and pulp processing industries at environmentally relevant levels [18,19], in the future, biopolymers from other bacteria and their composites can be promising materials for this; the *C*. *elegans* assay for assuring the safe levels of phytoestrogens may prove economical and practicable. In the present study, the *E*. *hirae* biopolymer proved to be completely safe, as demonstrated from the RAW 264.7 cell lines, and therefore suitable for applications in drinking water. To the best of our knowledge, potential microbial biopolymers aimed at removing phytoestrogen endocrine disruptors from water have not yet been reported. We are currently developing green composite materials using the biopolymer for removing endocrine disruptors in water.

## 5. Conclusions

Overall, the present study demonstrated the capabilities of extracellular polymers produced by *E. hirae* for binding and removing the phytoestrogens daidzein, biochanin A, formonetin and genistein. In vitro binding studies proved that the biopolymer could effectively remove all four phytoestrogens tested, albeit with varying capability. The good fit of the adsorption energy data to the Langmuir adsorption isotherms further corroborated these observations. Spatiotemporal effects in *C. elegans* challenged with phytoestrogens revealed complete protection in case of daidzein by the biopolymer. These results provide knowledge for developing more effective biopolymer composite materials for treating surface waters that have consistently high levels of discharges of phytoestrogens.

## Figures and Tables

**Table 1:** Bacterial sources of biopolymers and their phytoestrogen (PE)-binding ability. The PEs used were formononetin; BCA: biochanin A; G: genistein; and D: daidzein. The initial screening was performed with 1 mg of purified extracellular polymers from each isolate; the concentration of PEs was 100 µg/mL.

Biopolymer Producing Microorganisms	Biopolymer–PE Binding (%)	Isolation Source
*Enterococcus hirae* MG6	F(71%), G(72%), BCA(69%), D(87%)	Agro processing industry sludge
*Bacillus cereus*	F(54%), G(43%), BCA(25%), D(26%)	Legume farm soil
*Citrobacter brakii* MG303	F(14%), G(23%), BCA(45%), D(56%)	Alfalfa farm soil
*Bacillus cytotoxicus* MG303	F(54%), G(43%), BCA(35%), D(26%)	Soya processing industry effluent
*Enterococcus vikkiensis*	F(64%), G(53%), BCA(35%), D(81%)	Paper industry sludge

**Table 2:** Isothermal parameters of the phytoestrogens treated with the purified *E. hirae* biopolymer.

Phytoestrogens	Concentration	Qmax	K_L_	R^2^	R_L_
Formononetin	1.5 mg/mL	0.10	0.16	0.95	0.96
Genistein		0.32	0.63	0.97	0.62
Biochanin A		0.04	0.29	0.97	0.86
Daidzein	1 mg/mL	0.14	0.32	0.91	0.84

**Table 3:** (a) The effect of cytotoxicity of the *E. hirae* biopolymer on RAW264.7 cells. Cells were incubated with the purified biopolymer at doses ranging from 5 to 250 µg/mL at 37 °C for 48 h. Cell viability was measured by the MTT assay. (b) LC50 (lethal concentration) values of daidzein, genistein, biochanin A and formononetin on *C. elegans* after 24 h exposure. ND: Not detected Figures in parenthesis indicate the mean ± S.D (*n* = 3).

(a) Dose of Biopolymer (µg/mL)	Cell Viability (%)
0	99.6 ± 4.3
5	98.4 ± 6.4
10	98 ± 7.3
15	97.9 ± 7.4
20	98.3 ± 11.2
25	98.0 ± 9.7
50	97.8 ± 8.8
75	97.2 ± 9.3
100	96.7 ± 5.6
150	95.8 ± 4.3
200	96.3 ± 6.6
250	97.5 ± 9.6
**(b) Phytoestrogens Tested**	**LC50 (*C.elegens*)**
Daidzein (D)	100 µg/mL
Geneistein (G)	83 µg/mL
BiochaninA (BCA)	67 µg/mL
Formononetin (F)	53 µg/mL
Vehicle Control	ND

## Data Availability

Data of the experiments are available with the authors.

## Supplementary Materials

The following are available online at https://www.mdpi.com/article/10.3390/ijerph181910040/s1, Table S1. Compositional analysis of the *E.hirae* biopolymer.

## References

[1] Patisaul H.B., Jefferson W. (2010). The pros and cons of phytoestrogens. Front. Neuroendocrinol..

[2] Crain D.A., Janssen S.J., Edwards T.M., Heindel J., Ho S.-M., Hunt P., Iguchi T., Juul A., McLachlan J.A., Schwartz J. (2008). Female reproductive disorders: The roles of endocrine-disrupting compounds and developmental timing. Fertil. Steril..

[3] Thompson T.J., Briggs M.A., Phillips P.J., Blazer V.S., Smalling K.L., Kolpin D.W., Wagner T. (2021). Groundwater discharges as a source of phytoestrogens and other agriculturally derived contaminants to streams. Sci. Total Environ..

[4] Fukuhara T., Iwasaki S., Kawashima M., Shinohara O., Abe I. (2006). Adsorbability of estrone and 17β-estradiol in water onto activated carbon. Water Res..

[5] Urase T., Kikuta T. (2005). Separate estimation of adsorption and degradation of pharmaceutical substances and estrogens in the activated sludge process. Water Res..

[6] Caldwell D.J., Mastrocco F., Nowak E., Johnston J., Yekel H., Pfeiffer D., Hoyt M., DuPlessie B.M., Anderson P.D. (2010). An assessment of potential exposure and risk from estrogens in drinking water. Environ. Health Perspect..

[7] Khaira G.K., Ganguli A., Ghosh M. (2014). Antimicrobial efficacy and in vivo toxicity studies of a quaternized biopolymeric flocculant. J. Water Health.

[8] Sharma V., Ghosh M. (2021). Characterization of immunomodulatory, anticancer and antioxidant properties of an extracellular polymer produced by Enterococcus sp. in vegetable waste medium. Environ. Sustain..

[9] Zdunczyk Z., Juskiewicz J., Frejnagel S., Wroblewska M., Krefft B., Gulewicz K. Influence of Oligosaccharides from Lupin Seeds and Fructooligosaccharides on Utilisation of Protein by Rats and Absorption of Nutrients in the Small Intestine. Proceedings of the 9th International Lupin Conference.

[10] Sehgal S. (2013). Surface Assimilation of Phytoestrogens by Microbial Biopolymers. Master’s Thesis.

[11] Stiernagle T. (2006). Maintenance of C. elegans WormBook. The C. elegans research community. WormBook.

[12] Harlow P.H., Perry S.J., Widdison S., Daniels S., Bondo E., Lamberth C., Currie R.A., Flemming A.J. (2016). The nematode Caenorhabditis elegans as a tool to predict chemical activity on mammalian development and identify mechanisms influencing toxicological outcome. Sci. Rep..

[13] Jain S., Ghosh M. (2020). Effective removal of Bisphenol A from plastic waste leachates by microbial polymer impregnated with activated carbon. Int. J. Environ. Sci. Technol..

[14] Jahromi F.A., Moore F., Keshavarzi B., Mohebbi-Nozar S.L., Mohammadi Z., Sorooshian A., Abbasi S. (2020). Bisphenol A (BPA) and polycyclic aromatic hydrocarbons (PAHs) in the surface sediment and bivalves from Hormozgan Province coastline in the Northern Persian Gulf: A focus on source apportionment. Mar. Pollut. Bull..

[15] Gabet-Giraud V., Miege C., Choubert J., Ruel S.M., Coquery M. (2010). Occurrence and removal of estrogens and beta blockers by various processes in wastewater treatment plants. Sci. Total Environ..

[16] Hunt P.R., Olejnik N., Sprando R.L. (2012). Toxicity ranking of heavy metals with screening method using adult Caenorhabditis elegans and propidium iodide replicates toxicity ranking in rat. Food Chem. Toxicol..

[17] Kamaladevi A., Balamurugan K. (2015). Role of PMK-1/p38 MAPK defense in Caenorhabditis elegans against Klebsiella pneumoniae infection during host-pathogen interaction. Pathog. Dis..

[18] Lundgren M.S., Novak P.J. (2009). Quantification of phytoestrogens in industrial waste streams. Environ. Toxicol. Chem. Int. J..

[19] Patil D.V., Rallapalli P.B.S., Dangi G.P., Tayade R.J., Somani R.S., Bajaj H.C. (2011). MIL-53 (Al): An efficient adsorbent for the removal of nitrobenzene from aqueous solutions. Ind. Eng. Chem. Res..

