# Supplementary material for: Microbial Polymers as Sustainable Agents for Mitigating Health Risks of Plant-Based Endocrine Disruptors in Surface Water
Source: Int J Environ Res Public Health. 2021 Sep 24;18(19):10040. doi: 10.3390/ijerph181910040 (PMC8507969; doi:10.3390/ijerph181910040)
Supplement: Supplementary file 1 [file ijerph-18-10040-s001.zip › ijerph-1325918-SI.pdf]

Table S1. Compositional ananlysis of the *E.hirae* biopolymer.

Components	Composition(%)
Total Sugar	38.0
Hexoseamines	26.00
Uronic acid	8.70
Pyruvic acid	9.3
Proteins	18.6
